# 3,5,7-Trimeth­oxy-2-(4-methoxy­phen­yl)-4*H*-1-benzopyran-4-one

**DOI:** 10.1107/S1600536809040938

**Published:** 2009-10-10

**Authors:** Thammarat Aree, Pattara Sawasdee

**Affiliations:** a Department of Chemistry, Faculty of Science, Chulalongkorn University, Phyathai Road, Pathumwan, Bangkok 10330, Thailand, and The Center for Petroleum Petrochemicals and Advanced Materials, Chulalongkorn University, Bangkok 10330, Thailand

## Abstract

In the title compound, C_19_H_18_O_6_, also known as 3,4′,5,7-tetra­methoxy­flavone, the dihedral angle between the benzopyran-4-one group and the attached benzene ring is 11.23 (8)°. An intra­molecular C—H⋯O hydrogen bond generates an *S*(6) ring motif. In the crystal, mol­ecules are linked into a two-dimensional network parallel to (0
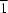
1) by inter­molecular C—H⋯O hydrogen bonds, which generate *R*_4_^4^(20), *R*_4_^4^(12) and *R*_2_^2^(14) ring motifs. Adjacent networks interact by π–π inter­actions between the pyran ring and its methoxy­phenyl substituent [centroid–centroid distance = 3.5267 (8) Å].

## Related literature

For related structures, see: Aree *et al.* (2009 ▶) and the Cambridge Structural Database [Allen (2002 ▶); Bruno *et al.* (2002 ▶)]. For the graph-set description of hydrogen-bond patterns, see: Bernstein *et al.* (1995 ▶).
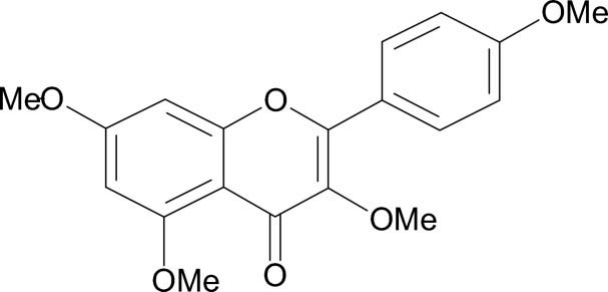


## Experimental

### 

#### Crystal data

C_19_H_18_O_6_
*M* *_r_* = 342.33
Triclinic, 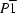

*a* = 8.7854 (3) Å
*b* = 9.2743 (4) Å
*c* = 10.6950 (4) Å
α = 70.749 (1)°
β = 81.448 (1)°
γ = 83.078 (1)°
*V* = 811.15 (5) Å^3^
*Z* = 2
Mo *K*α radiation
μ = 0.11 mm^−1^
*T* = 298 K
0.40 × 0.22 × 0.18 mm

#### Data collection

Bruker SMART APEXII CCD area-detector diffractometer
Absorption correction: multi-scan (**SADABS**; Bruker, 2005 ▶) *T*_min_ = 0.839, *T*_max_ = 0.946
5901 measured reflections
3930 independent reflections
2827 reflections with *I* > 2σ(*I*)
*R*_int_ = 0.023

#### Refinement

*R*[*F* ^2^ > 2σ(*F* ^2^)] = 0.044
*wR*(*F* ^2^) = 0.131
*S* = 1.06
3930 reflections
230 parameters
H-atom parameters constrained
Δρ_max_ = 0.23 e Å^−3^
Δρ_min_ = −0.19 e Å^−3^

### 

Data collection: *APEX2* (Bruker, 2005 ▶); cell refinement: *SAINT* (Bruker, 2005 ▶); data reduction: *SAINT*; program(s) used to solve structure: *SHELXTL* (Sheldrick, 2008 ▶); program(s) used to refine structure: *SHELXTL*; molecular graphics: *ORTEP-3* (Farrugia, 1997 ▶) and *Mercury* (Macrae *et al.* 2006 ▶).; software used to prepare material for publication: *SHELXTL*.

## Supplementary Material

Crystal structure: contains datablocks I, global. DOI: 10.1107/S1600536809040938/ci2932sup1.cif

Structure factors: contains datablocks I. DOI: 10.1107/S1600536809040938/ci2932Isup2.hkl

Additional supplementary materials:  crystallographic information; 3D view; checkCIF report

## Figures and Tables

**Table 1:** Hydrogen-bond geometry (Å, °)

*D*—H⋯*A*	*D*—H	H⋯*A*	*D*⋯*A*	*D*—H⋯*A*
C15—H15⋯O5	0.93	2.23	2.8690 (18)	126
C17—H17*B*⋯O2^i^	0.96	2.48	3.2674 (19)	139
C17—H17*B*⋯O3^i^	0.96	2.61	3.458 (2)	148
C18—H18*B*⋯O6^ii^	0.96	2.57	3.530 (2)	173
C19—H19*C*⋯O5^iii^	0.96	2.51	3.457 (2)	170

Symmetry codes: (i) 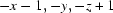; (ii) 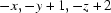; (iii) 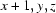.

**Fig. 1.**
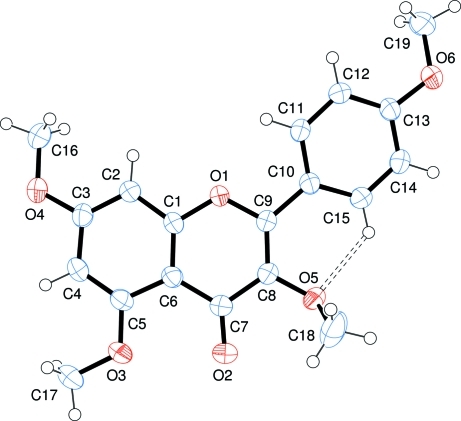
The molecular structure of (I), with atom numbering and 50% probability displacement ellipsoids. An intramolecular C—H···O hydrogen bond forming an S(6) motif is shown as a dashed line.

**Fig. 2.**
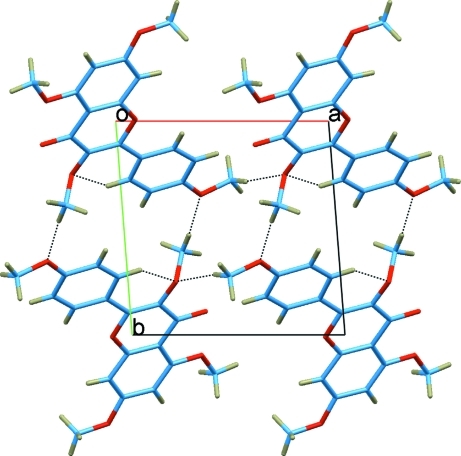
Part of a ribbon formed by intermolecular C—H···O hydrogen bonds, with R44(20) and R44(12) ring motifs. Hydrogen bonds are shown as dashed lines.

**Fig. 3.**
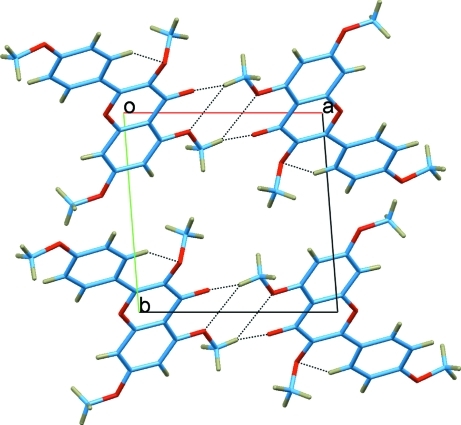
A view of R22(14) ring motifs which connect adjacent ribbons. Hydrogen bonds are shown as dashed lines.

**Fig. 4.**
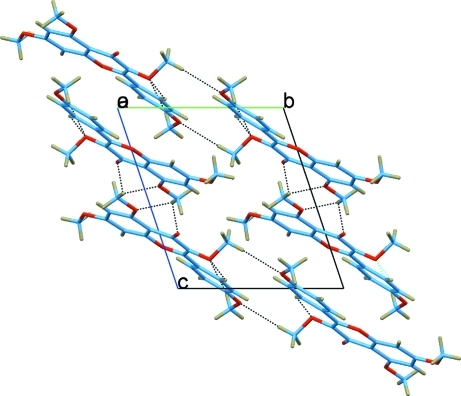
Part of the crystal structure of (I), showing the stacking of pyran and 4-methoxyphenyl rings. Hydrogen bonds are shown as dashed lines.

## supplementary crystallographic information

### Comment

The title compound, (I), (3,5,7-trimethoxy-2-(4-methoxyphenyl)-4*H*- 1-benzopyran-4-one or 3,4',5,7-tetramethoxyflavone), (Fig.1), is a secondary metabolite that was isolated from a Thai medicinal plant, *Kaempferia parviflora*. Several flavones have also been isolated from this plant and their crystal structures have been reported, for example see Aree *et al.* (2009) and references cited therein. Here, we report the crystal structure of another flavone in an anhydrous form having no strong hydrogen bond donor. Weak C—H···O hydrogen bonds play a key role in stabilizing the crystal lattice.

The molecular structure of (I) deviates from a planar geometry; the interplanar angle between the benzopyran-4-one group and the attached phenyl group is 11.23 (8)° (Fig. 1). A search in the Cambridge Structural Database [Version 1.11 (Allen, 2002); CONQUEST (Bruno *et al.*, 2002)] indicate that this feature is frequently observed . The three methoxy C16, C17 and C19 atoms slightly deviate from the mean planes of the attached benzopyran or phenyl rings by 0.288 (3), -0.119 (3) and 0.355 (3) Å whereas atom C18 deviates from the benzopyran plane by -0.933 (3) Å. The corresponding values of torsion angles are C16—O4—C3—C2 = 3.0 (2)°, C17—O3—C5—C4 = 0.4 (2)°, C19—O6—C13—C12 = -15.9 (2)° and C18—O5—C8—C9 = 111.09 (17)°. The flavone molecule is stabilized by an intramolecular C15—H···O5 hydrogen bond that generates an *S*(6) ring motif (Bernstein *et al.*, 1995).

In the crystal, molecules are linked to form a ribbon-like structure by intermolecular C18—H18B···O6^ii^ and C19—H19C···.O5^iii^ hydrogen bonds, generating *R*_2_^2^(20) and *R*_4_^4^(12) ring motifs (Bernstein *et al.*, 1995) (Fig. 2). The adjacent inversion-related ribbons are cross-linked into a two-dimensional network parallel to the (011) by intermolecular C17—H17B···O2^i^ and C17—H17B···O3^i^ hydrogen bonds, generating *R*_2_^2^(14) ring motifs (Bernstein *et al.*, 1995) (Fig. 3). The crystal structure is further stabilized by π–π interactions (Fig. 4) between O1/C1/C6-C9 and C10-C15 rings of the molecules in adjacent networks, with a centroid-to-centroid distance of 3.5267 (8) Å.

### Experimental

The title compound, (I), was extracted from *Kaempferia parviflora*, a medicinal plant from the north-east of Thailand. Single crystals of (I) were obtained by slow evaporation of a methanol–water (1:1, *v*/*v*) solution at room temperature.

### Refinement

All H atoms were located in a difference map and then refined using a riding model, with C-H = 0.93 Å (aromatic) and *U*_iso_(H) = 1.2*U*_eq_(C), and C-H = 0.96 Å (methyl) and *U*_iso_(H) = 1.5*U*_eq_(C_methyl_).

### Crystal data

**Table:**

C_19_H_18_O_6_	*Z* = 2
*M**_r_* = 342.33	*F*(000) = 360
Triclinic, *P*1	*D*_x_ = 1.402 Mg m^−^^3^
Hall symbol: -P 1	Melting point: not measured K
*a* = 8.7854 (3) Å	Mo *K*α radiation, λ = 0.71073 Å
*b* = 9.2743 (4) Å	Cell parameters from 2649 reflections
*c* = 10.6950 (4) Å	θ = 2.9–29.0°
α = 70.749 (1)°	µ = 0.11 mm^−^^1^
β = 81.448 (1)°	*T* = 298 K
γ = 83.078 (1)°	Block, colourless
*V* = 811.15 (5) Å^3^	0.40 × 0.22 × 0.18 mm

### Data collection

**Table:**

Bruker SMART APEXII CCD area-detector diffractometer	3930 independent reflections
Radiation source: fine-focus sealed tube	2827 reflections with *I* > 2σ(*I*)
graphite	*R*_int_ = 0.023
φ and ω scans	θ_max_ = 28.3°, θ_min_ = 2.3°
Absorption correction: multi-scan (*SADABS*; Bruker, 2005)	*h* = −11→11
*T*_min_ = 0.839, *T*_max_ = 0.946	*k* = −12→12
5901 measured reflections	*l* = −10→14

### Refinement

**Table:**

Refinement on *F*^2^	Primary atom site location: structure-invariant direct methods
Least-squares matrix: full	Secondary atom site location: difference Fourier map
*R*[*F*^2^ > 2σ(*F*^2^)] = 0.044	Hydrogen site location: inferred from neighbouring sites
*wR*(*F*^2^) = 0.131	H-atom parameters constrained
*S* = 1.06	*w* = 1/[σ^2^(*F*_o_^2^) + (0.0642*P*)^2^ + 0.0796*P*] where *P* = (*F*_o_^2^ + 2*F*_c_^2^)/3
3930 reflections	(Δ/σ)_max_ = 0.001
230 parameters	Δρ_max_ = 0.23 e Å^−^^3^
0 restraints	Δρ_min_ = −0.19 e Å^−^^3^

### Special details

**Table:**

Geometry. All e.s.d.'s (except the e.s.d. in the dihedral angle between two l.s. planes) are estimated using the full covariance matrix. The cell e.s.d.'s are taken into account individually in the estimation of e.s.d.'s in distances, angles and torsion angles; correlations between e.s.d.'s in cell parameters are only used when they are defined by crystal symmetry. An approximate (isotropic) treatment of cell e.s.d.'s is used for estimating e.s.d.'s involving l.s. planes.
Refinement. Refinement of *F*^2^ against ALL reflections. The weighted *R*-factor *wR* and goodness of fit *S* are based on *F*^2^, conventional *R*-factors *R* are based on *F*, with *F* set to zero for negative *F*^2^. The threshold expression of *F*^2^ > σ(*F*^2^) is used only for calculating *R*-factors(gt) *etc*. and is not relevant to the choice of reflections for refinement. *R*-factors based on *F*^2^ are statistically about twice as large as those based on *F*, and *R*- factors based on ALL data will be even larger.

### Fractional atomic coordinates and isotropic or equivalent isotropic displacement parameters (Å^2^)

**Table:**

	*x*	*y*	*z*	*U*_iso_*/*U*_eq_	
O1	0.09284 (11)	−0.00853 (11)	0.77045 (10)	0.0384 (2)	
O2	−0.35336 (13)	0.11019 (15)	0.69876 (14)	0.0627 (4)	
O3	−0.31863 (12)	−0.09508 (12)	0.56603 (11)	0.0472 (3)	
O4	0.14430 (12)	−0.41488 (12)	0.58844 (11)	0.0491 (3)	
O5	−0.22549 (11)	0.24963 (11)	0.83872 (10)	0.0417 (3)	
O6	0.37277 (13)	0.34385 (13)	1.08046 (12)	0.0554 (3)	
C1	0.02711 (15)	−0.08612 (15)	0.70567 (13)	0.0334 (3)	
C2	0.12382 (16)	−0.20556 (15)	0.67915 (14)	0.0370 (3)	
H2	0.2237	−0.2266	0.7030	0.044*	
C3	0.06590 (16)	−0.29116 (15)	0.61632 (13)	0.0363 (3)	
C4	−0.08153 (16)	−0.25525 (16)	0.57554 (14)	0.0377 (3)	
H4	−0.1168	−0.3118	0.5298	0.045*	
C5	−0.17492 (16)	−0.13643 (16)	0.60281 (13)	0.0358 (3)	
C6	−0.12267 (15)	−0.04848 (15)	0.67302 (13)	0.0337 (3)	
C7	−0.21732 (16)	0.07127 (16)	0.71647 (14)	0.0381 (3)	
C8	−0.13869 (16)	0.14406 (16)	0.78818 (13)	0.0343 (3)	
C9	0.01016 (15)	0.10572 (15)	0.81213 (13)	0.0329 (3)	
C10	0.10563 (15)	0.16951 (15)	0.88037 (13)	0.0334 (3)	
C11	0.26309 (17)	0.12727 (17)	0.87961 (14)	0.0397 (3)	
H11	0.3069	0.0596	0.8342	0.048*	
C12	0.35723 (17)	0.18231 (17)	0.94411 (15)	0.0415 (3)	
H12	0.4622	0.1522	0.9415	0.050*	
C13	0.29333 (17)	0.28240 (16)	1.01227 (14)	0.0383 (3)	
C14	0.13743 (18)	0.32664 (18)	1.01423 (16)	0.0464 (4)	
H14	0.0944	0.3944	1.0597	0.056*	
C15	0.04512 (17)	0.27193 (18)	0.94994 (16)	0.0446 (4)	
H15	−0.0596	0.3034	0.9526	0.054*	
C16	0.2918 (2)	−0.4624 (2)	0.6324 (2)	0.0593 (5)	
H16A	0.3609	−0.3837	0.5873	0.089*	
H16B	0.3308	−0.5549	0.6130	0.089*	
H16C	0.2838	−0.4808	0.7268	0.089*	
C17	−0.3753 (2)	−0.1803 (2)	0.49641 (19)	0.0556 (4)	
H17A	−0.3085	−0.1736	0.4153	0.083*	
H17B	−0.4775	−0.1393	0.4756	0.083*	
H17C	−0.3781	−0.2856	0.5512	0.083*	
C18	−0.2656 (3)	0.3918 (2)	0.74252 (19)	0.0718 (6)	
H18A	−0.1763	0.4263	0.6807	0.108*	
H18B	−0.3027	0.4663	0.7866	0.108*	
H18C	−0.3449	0.3787	0.6952	0.108*	
C19	0.5210 (2)	0.2757 (2)	1.11183 (18)	0.0582 (5)	
H19A	0.5135	0.1701	1.1643	0.087*	
H19B	0.5620	0.3284	1.1615	0.087*	
H19C	0.5882	0.2825	1.0309	0.087*	

### Atomic displacement parameters (Å^2^)

**Table:**

	*U*^11^	*U*^22^	*U*^33^	*U*^12^	*U*^13^	*U*^23^
O1	0.0329 (5)	0.0393 (5)	0.0537 (6)	0.0034 (4)	−0.0130 (4)	−0.0279 (5)
O2	0.0377 (6)	0.0752 (8)	0.0972 (9)	0.0149 (6)	−0.0282 (6)	−0.0546 (7)
O3	0.0394 (6)	0.0499 (6)	0.0641 (7)	0.0004 (5)	−0.0220 (5)	−0.0281 (5)
O4	0.0479 (6)	0.0465 (6)	0.0663 (7)	0.0043 (5)	−0.0132 (5)	−0.0356 (5)
O5	0.0376 (5)	0.0459 (6)	0.0466 (5)	0.0098 (4)	−0.0091 (4)	−0.0241 (5)
O6	0.0479 (6)	0.0617 (7)	0.0775 (8)	0.0096 (5)	−0.0264 (6)	−0.0467 (6)
C1	0.0337 (7)	0.0329 (7)	0.0381 (6)	−0.0042 (5)	−0.0073 (5)	−0.0154 (5)
C2	0.0345 (7)	0.0358 (7)	0.0451 (7)	0.0007 (6)	−0.0100 (6)	−0.0178 (6)
C3	0.0394 (8)	0.0324 (7)	0.0391 (7)	−0.0028 (6)	−0.0032 (6)	−0.0147 (6)
C4	0.0409 (8)	0.0375 (7)	0.0406 (7)	−0.0085 (6)	−0.0080 (6)	−0.0169 (6)
C5	0.0341 (7)	0.0367 (7)	0.0384 (7)	−0.0062 (6)	−0.0086 (6)	−0.0111 (6)
C6	0.0337 (7)	0.0315 (7)	0.0378 (7)	−0.0034 (5)	−0.0075 (5)	−0.0117 (5)
C7	0.0331 (7)	0.0391 (7)	0.0454 (7)	0.0000 (6)	−0.0095 (6)	−0.0167 (6)
C8	0.0334 (7)	0.0351 (7)	0.0366 (6)	0.0014 (5)	−0.0048 (5)	−0.0155 (5)
C9	0.0329 (7)	0.0318 (7)	0.0364 (6)	0.0004 (5)	−0.0048 (5)	−0.0148 (5)
C10	0.0351 (7)	0.0327 (7)	0.0351 (6)	−0.0007 (5)	−0.0075 (5)	−0.0136 (5)
C11	0.0393 (8)	0.0406 (8)	0.0473 (7)	0.0071 (6)	−0.0112 (6)	−0.0257 (6)
C12	0.0337 (7)	0.0463 (8)	0.0521 (8)	0.0076 (6)	−0.0135 (6)	−0.0256 (7)
C13	0.0408 (8)	0.0378 (7)	0.0427 (7)	0.0007 (6)	−0.0137 (6)	−0.0188 (6)
C14	0.0447 (9)	0.0502 (9)	0.0562 (9)	0.0083 (7)	−0.0116 (7)	−0.0347 (7)
C15	0.0339 (7)	0.0543 (9)	0.0563 (9)	0.0069 (7)	−0.0111 (6)	−0.0329 (7)
C16	0.0516 (10)	0.0537 (10)	0.0871 (13)	0.0130 (8)	−0.0179 (9)	−0.0436 (9)
C17	0.0490 (10)	0.0620 (10)	0.0707 (11)	−0.0037 (8)	−0.0254 (8)	−0.0333 (9)
C18	0.1032 (16)	0.0453 (10)	0.0648 (11)	0.0232 (10)	−0.0173 (11)	−0.0217 (9)
C19	0.0463 (9)	0.0770 (12)	0.0683 (11)	0.0066 (9)	−0.0231 (8)	−0.0427 (10)

### Geometric parameters (Å, °)

**Table:**

O1—C9	1.3707 (15)	C10—C11	1.3912 (19)
O1—C1	1.3708 (15)	C10—C15	1.4018 (19)
O2—C7	1.2300 (17)	C11—C12	1.3871 (19)
O3—C5	1.3517 (16)	C11—H11	0.93
O3—C17	1.4212 (17)	C12—C13	1.381 (2)
O4—C3	1.3616 (16)	C12—H12	0.93
O4—C16	1.4150 (19)	C13—C14	1.381 (2)
O5—C8	1.3707 (16)	C14—C15	1.372 (2)
O5—C18	1.422 (2)	C14—H14	0.93
O6—C13	1.3657 (16)	C15—H15	0.93
O6—C19	1.4141 (19)	C16—H16A	0.96
C1—C6	1.3860 (18)	C16—H16B	0.96
C1—C2	1.3921 (18)	C16—H16C	0.96
C2—C3	1.3753 (18)	C17—H17A	0.96
C2—H2	0.93	C17—H17B	0.96
C3—C4	1.395 (2)	C17—H17C	0.96
C4—C5	1.377 (2)	C18—H18A	0.96
C4—H4	0.93	C18—H18B	0.96
C5—C6	1.4270 (18)	C18—H18C	0.96
C6—C7	1.4641 (19)	C19—H19A	0.96
C7—C8	1.4603 (19)	C19—H19B	0.96
C8—C9	1.3526 (18)	C19—H19C	0.96
C9—C10	1.4740 (18)		
			
C9—O1—C1	121.22 (10)	C10—C11—H11	118.8
C5—O3—C17	117.68 (12)	C13—C12—C11	119.26 (13)
C3—O4—C16	117.79 (11)	C13—C12—H12	120.4
C8—O5—C18	115.26 (12)	C11—C12—H12	120.4
C13—O6—C19	118.35 (12)	O6—C13—C14	115.45 (12)
O1—C1—C6	122.08 (12)	O6—C13—C12	125.06 (13)
O1—C1—C2	113.58 (11)	C14—C13—C12	119.50 (12)
C6—C1—C2	124.33 (12)	C15—C14—C13	120.90 (13)
C3—C2—C1	117.38 (12)	C15—C14—H14	119.6
C3—C2—H2	121.3	C13—C14—H14	119.6
C1—C2—H2	121.3	C14—C15—C10	121.25 (13)
O4—C3—C2	124.15 (13)	C14—C15—H15	119.4
O4—C3—C4	114.65 (12)	C10—C15—H15	119.4
C2—C3—C4	121.20 (13)	O4—C16—H16A	109.5
C5—C4—C3	120.26 (12)	O4—C16—H16B	109.5
C5—C4—H4	119.9	H16A—C16—H16B	109.5
C3—C4—H4	119.9	O4—C16—H16C	109.5
O3—C5—C4	123.43 (12)	H16A—C16—H16C	109.5
O3—C5—C6	115.93 (12)	H16B—C16—H16C	109.5
C4—C5—C6	120.63 (12)	O3—C17—H17A	109.5
C1—C6—C5	116.09 (12)	O3—C17—H17B	109.5
C1—C6—C7	119.08 (12)	H17A—C17—H17B	109.5
C5—C6—C7	124.77 (12)	O3—C17—H17C	109.5
O2—C7—C8	120.11 (13)	H17A—C17—H17C	109.5
O2—C7—C6	125.17 (13)	H17B—C17—H17C	109.5
C8—C7—C6	114.71 (11)	O5—C18—H18A	109.5
C9—C8—O5	119.91 (11)	O5—C18—H18B	109.5
C9—C8—C7	123.11 (12)	H18A—C18—H18B	109.5
O5—C8—C7	116.87 (11)	O5—C18—H18C	109.5
C8—C9—O1	119.68 (11)	H18A—C18—H18C	109.5
C8—C9—C10	129.53 (12)	H18B—C18—H18C	109.5
O1—C9—C10	110.79 (10)	O6—C19—H19A	109.5
C11—C10—C15	116.66 (12)	O6—C19—H19B	109.5
C11—C10—C9	120.31 (12)	H19A—C19—H19B	109.5
C15—C10—C9	123.02 (12)	O6—C19—H19C	109.5
C12—C11—C10	122.43 (13)	H19A—C19—H19C	109.5
C12—C11—H11	118.8	H19B—C19—H19C	109.5
			
C9—O1—C1—C6	3.33 (19)	C18—O5—C8—C7	−72.50 (18)
C9—O1—C1—C2	−175.58 (12)	O2—C7—C8—C9	179.37 (14)
O1—C1—C2—C3	178.92 (12)	C6—C7—C8—C9	0.6 (2)
C6—C1—C2—C3	0.0 (2)	O2—C7—C8—O5	3.1 (2)
C16—O4—C3—C2	3.0 (2)	C6—C7—C8—O5	−175.74 (11)
C16—O4—C3—C4	−177.12 (14)	O5—C8—C9—O1	174.86 (11)
C1—C2—C3—O4	−177.39 (13)	C7—C8—C9—O1	−1.3 (2)
C1—C2—C3—C4	2.8 (2)	O5—C8—C9—C10	−4.9 (2)
O4—C3—C4—C5	177.35 (12)	C7—C8—C9—C10	178.90 (13)
C2—C3—C4—C5	−2.8 (2)	C1—O1—C9—C8	−0.58 (19)
C17—O3—C5—C4	0.4 (2)	C1—O1—C9—C10	179.24 (11)
C17—O3—C5—C6	−179.72 (13)	C8—C9—C10—C11	−171.67 (14)
C3—C4—C5—O3	179.81 (12)	O1—C9—C10—C11	8.53 (18)
C3—C4—C5—C6	0.0 (2)	C8—C9—C10—C15	9.3 (2)
O1—C1—C6—C5	178.53 (12)	O1—C9—C10—C15	−170.51 (13)
C2—C1—C6—C5	−2.7 (2)	C15—C10—C11—C12	0.1 (2)
O1—C1—C6—C7	−4.0 (2)	C9—C10—C11—C12	−178.98 (13)
C2—C1—C6—C7	174.78 (13)	C10—C11—C12—C13	0.2 (2)
O3—C5—C6—C1	−177.23 (12)	C19—O6—C13—C14	164.09 (15)
C4—C5—C6—C1	2.63 (19)	C19—O6—C13—C12	−15.9 (2)
O3—C5—C6—C7	5.5 (2)	C11—C12—C13—O6	179.50 (14)
C4—C5—C6—C7	−174.66 (13)	C11—C12—C13—C14	−0.4 (2)
C1—C6—C7—O2	−176.71 (14)	O6—C13—C14—C15	−179.64 (14)
C5—C6—C7—O2	0.5 (2)	C12—C13—C14—C15	0.3 (2)
C1—C6—C7—C8	2.05 (19)	C13—C14—C15—C10	0.0 (3)
C5—C6—C7—C8	179.26 (12)	C11—C10—C15—C14	−0.3 (2)
C18—O5—C8—C9	111.09 (17)	C9—C10—C15—C14	178.81 (14)

### Hydrogen-bond geometry (Å, °)

**Table:**

*D*—H···*A*	*D*—H	H···*A*	*D*···*A*	*D*—H···*A*
C15—H15···O5	0.93	2.23	2.8690 (18)	126
C17—H17B···O2^i^	0.96	2.48	3.2674 (19)	139
C17—H17B···O3^i^	0.96	2.61	3.458 (2)	148
C18—H18B···O6^ii^	0.96	2.57	3.530 (2)	173
C19—H19C···O5^iii^	0.96	2.51	3.457 (2)	170

Symmetry codes: (i) −*x*−1, −*y*, −*z*+1; (ii) −*x*, −*y*+1, −*z*+2; (iii) *x*+1, *y*, *z*.

## References

[bb1] Allen, F. H. (2002). *Acta Cryst.* B**58**, 380–388. 10.1107/s0108768102003890 12037359

[bb2] Aree, T., Sabphon, C. & Sawasdee, P. (2009). *Acta Cryst.* E**65**, o2693. 10.1107/S1600536809040513 PMC2971445 21578297

[bb3] Bernstein, J., Davis, R. E., Shimoni, L. & Chang, N.-L. (1995). *Angew. Chem. Int. Ed. Engl.* **34**, 1555–1573.

[bb4] Bruker (2005). *APEX2*, *SAINT* and *SADABS* Bruker AXS Inc., Madison, Wisconsin, USA.

[bb5] Bruno, I. J., Cole, J. C., Edgington, P. R., Kessler, M., Macrae, C. F., McCabe, P., Pearson, J. & Taylor, R. (2002). *Acta Cryst.* B**58**, 389–397. 10.1107/s0108768102003324 12037360

[bb6] Farrugia, L. J. (1997). *J. Appl. Cryst.* **30**, 565.

[bb7] Macrae, C. F., Edgington, P. R., McCabe, P., Pidcock, E., Shields, G. P., Taylor, R., Towler, M. & van de Streek, J. (2006). *J. Appl. Cryst.* **39**, 453–457.

[bb8] Sheldrick, G. M. (2008). *Acta Cryst.* A**64**, 112–122. 10.1107/S0108767307043930 18156677

